# Cannabis-Derived Product Types, Flavors, and Compound Types From an E-Commerce Website

**DOI:** 10.1001/jamanetworkopen.2024.40376

**Published:** 2024-10-21

**Authors:** Matthew C. Nali, Joshua S. Yang, Zhuoran Li, Meng Zhen Larsen, Tim K. Mackey

**Affiliations:** 1 San Diego Supercomputer Center, University of California, San Diego; 2 Global Health Policy and Data Institute, San Diego, California; 3 S-3 Research, San Diego, California; 4 Department of Public Health, California State University, Fullerton; 5 Global Health Program, Department of Anthropology, University of California, San Diego

## Abstract

**Importance:**

Cannabis-derived products (CDPs) are widely available and diverse. A classification of product and flavor types is necessary to establish a foundation for comparative research, although research aiming to classify cannabis in its variety of products, flavors, and cannabinoid compounds based on public online e-commerce data is lacking.

**Objective:**

To analyze data from a large cannabis e-commerce marketplace to identify and characterize cannabis product types, flavors, and compound types.

**Design, Setting, and Participants:**

This qualitative study was conducted in 2 phases: (1) data mining of the cannabis e-commerce website Weedmaps for product listings in the US between September 1 and November 30, 2023 and (2) grouping CDPs into product, flavor, and cannabinoid compound categories.

**Exposures:**

Cannabis product listings.

**Main Outcomes and Measures:**

Product listings and descriptions were extracted from the platform. Coding was performed for specific product characteristics, routes of administration (ROAs), and characterization of flavors.

**Results:**

A total of 573 854 unique US CDP sales listings from the platform were collected; after removing 72 842 nonconsumable items, 501 012 were analyzed. Product ROAs included multisystem (205 637 [41.04%]), respiratory (185 296 [36.98%]), digestive (98 941 [19.75%]), epidermal (9487 [1.89%]), and oral (1651 [0.33%]). Nearly half (210 575 [42.03%]) of all product listings included at least 1 flavor, with 247 762 instances of flavors. The 3 most common flavors were lemon (22 106 [8.92%]), cake (19 463 [7.86%]), and strawberry (13 961 [5.63%]). The most common cannabinoid compound type was Δ^9^-tetrahydrocannabinol (54 699 [63.30%]).

**Conclusions and Relevance:**

This qualitative study categorized more than half a million CDPs for product and flavor types. Results are needed for comparative studies on product and market availability and can help in assessing concerns about appealing characteristics. The results can also inform future market surveillance efforts aimed at identifying new and emerging products as cannabis policy continues to move toward greater legalization.

## Key Points

**Question:**

What types of cannabis products and their respective flavors are available on a cannabis e-commerce website?

**Findings:**

This qualitative study of 501 012 unique cannabis product listings sold in the US found that the top 3 routes of administration were multisystem (41%), respiratory (37%), and digestive (20%). The most common product categories were flower buds (20%), cartridges (12%), and joints (8%); the most common cannabinoid compound type was Δ^9^-tetrahydrocannabinol (63%); and among all products, 42% had at least 1 of 260 unique flavors.

**Meaning:**

Existing, new, and emerging characteristics of cannabis-derived products should be properly monitored and assessed for possible regulation and appropriate restrictions to ensure current and future safe and responsible adult use.

## Teaser

This qualitative study develops a schema to classify and describe the breadth of cannabis products commercially available in the US.

## Introduction

The policy environment regulating cannabis (including marijuana and hemp) has changed dramatically in a short period of time.^1,2^ As of February 2024, 24 US states had legalized nonmedical (ie, recreational) and medical cannabis use, with an additional 14 states permitting medical marijuana only, a rapid rise since Colorado and Washington first legalized recreational use of marijuana in 2012.^3,4^ Although cannabis remains a Schedule I substance under the Federal Controlled Substance Act, where any form of cultivation, distribution, and/or possession remains illegal, the Agricultural Improvement Act (also known as the 2018 Farm Bill) excluded hemp and cannabis derivates (eg, cannabis products with tetrahydrocannabinol [THC] under 0.3% on a dry basis), leading to new types of derived psychoactive cannabis products being sold in the US.^5,6,7,8^

Research suggests that cannabis legalization may be positively associated with cannabis use and cannabis use disorder.^9^ Cannabis is the most commonly used illicit drug in the US, with 50.7% of US residents 12 years and older reporting lifetime cannabis use, 24.9% reporting past year use, and 16.5% reporting past month use in 2022, all increases over similar patterns in 2021 and reflecting an increase in cannabis use among adults over time.^9,10^ While other drug and alcohol use among adolescents has been trending downward in recent decades, prevalence of cannabis use has fluctuated at similar or increased levels in the same period of time.^11^ Specific to young adults, the Monitoring the Future survey^12^ reported that in 2022, among younger adults aged 19 to 30 years, those reporting past-year and daily cannabis use had reached the highest levels ever.

One avenue where cannabis legalization may impact use is expansion of not only the number of products available but also the types and appeal of products through market innovations. Products vary by route of administration (ROAs) (eg, oral, inhaled), potency, compound composition (eg, Δ^9^-THC, cannabidiol, cannabichromene), characterizing features (eg, flavoring), and product type (eg, flower, concentrate).^6,13^ Product variability is reflected in an expanding array of cannabis-derived products (CDP) sold in online and physical retail settings and consumed among diverse cannabis users.^6,14,15,16,17^ Over time, the type, mix, and price of cannabis products purchased can vary in a specific locale.^18^

Even as the cannabis product market expands and diversifies, the policy environment remains fragmented, and the regulatory infrastructure is still maturing, posing ongoing concerns over product constituents and safety, labeling and health claims, and other unregulated marketing activities.^19,20,21,22,23,24,25,26,27,28,29^ A first step in improved product regulation is a more complete understanding of the cannabis product environment. However, no public health surveillance systems in the US collect data at a level of granularity necessary to properly evaluate and better inform cannabis products regulation.^17^ While some studies have begun to examine the CDP marketplace in greater detail, they have been limited in their geographic scope or smaller sample size.^6,15,17^

The growing popularity of national, online cannabis e-commerce outlets that connect consumers with cannabis products, retailers, and dispensaries provides an opportunity for big data market surveillance to develop a product taxonomy to characterize CDP across domains such as product type, flavor, and cannabinoid composition.^30^ Weedmaps is one of the largest and most popular cannabis e-commerce platforms, with an estimated 16.4 million monthly active users in 2022 and $188 million in revenue for 2023.^31,32^ This qualitative study combines approaches in data science and qualitative research to develop a schema to classify and describe the breadth of cannabis products commercially available in the US as marketed on a large e-commerce cannabis platform.

## Methods

Data analysis was carried out in 2 phases: (1) automated data collection from the platform using custom data mining approaches; and (2) manual annotation and content analysis to identify and categorize product characteristics and flavor types from cannabis product listings in the US. The reporting of the study follows the Standards for Reporting Qualitative Research (SRQR) guidelines. This study falls under a larger project examining new and emerging cannabis products with approval of the California State University, Fullerton, institutional review board, which exempts from review research that does not include data on human participants and does not required consent for the use of publicly available deidentified data.

### Data Collection

Weedmaps is one of the largest and most popular cannabis e-commerce platforms, with an estimated 16.4 million monthly active users in 2022 and $188 million in revenue for 2023.^31,32^ Market surveillance of CDP listings was conducted on the platform using a customized data mining approach from September 1 to November 30, 2023. As the platform is primarily a map-based web e-commerce site, a typical internet user visiting the site would have multiple options for searching for cannabis products, brands, and locations in the structured search query function available on the site (eg, searching for a product type or by location). Our data collection and sampling strategy mimicked how a user might search for cannabis products by generating structured automated searches using a custom data mining program developed in Python, version.3.8 (Python), for all cannabis products that appeared when searching for specific US-based locations. This was accomplished by dividing the US into 3 main regions for data collection—the contiguous US, the state of Alaska, and the state of Hawaii—and collecting all product listings that were returned based on a map-based grid search on multiple sets of geo coordinates covering the entire US. After product listings were collected, the Python package beautiful soup 4, version 4.6.0, was used to decode the hypertext markup language content to extract all available product attribute information from the platform’s product metadata (see eMethods in Supplement 1 for list of product attributes).

### Statistical Analysis

#### Manual Annotation and Content Analysis

Information for all CDP listings collected was manually entered into a database of product attributes from fields extracted from the platform. Initial grouping of CDPs into the classification schemas as reported below was performed through consensus coding with 2 study authors (M.C.N. and M.Z.L.) each coding all of the data and resolving conflicts through discussion. For flavor groupings, an inductive coding scheme was created through iterative coding, code creation and revision, and discussion among the coders with multiple reviews by a third study author (T.K.M.) after significant revisions. The final iteration of the inductive coding scheme was compared against the European Food Safety Authority FoodEx2 classification system (FoodEx2) by 3 study authors (M.C.N., J.S.Y., and T.K.M.).^33^ Categories and definitions from FoodEx2 were substituted for inductively created categories for the final codebook through a consensus process to minimize error and bias; some flavor classes were adapted from the FoodEx2 classification where applicable, while others were created inductively (see eTable 1 in Supplement 1). Products were grouped into flavor categories through team-based (M.C.N., J.S.Y., and T.K.M.) assignment, with a majority agreement used to resolve disagreements.

A similar process was used for the CDP product type grouping, with greater use of inductive classification because fewer applicable existing classification schema exist for adaptation to CDP product types.^34^ Two authors (M.C.N. and M.Z.L.) developed an inductively derived coding scheme through iterative coding, code creation and revision, and discussion. A second set of authors (M.C.N., J.S.Y., and T.K.M.) identified existing classification schema that could be applied to product type grouping and, through a consensus process, developed and finalized a codebook using existing resources where available.^34,35^ An inductive schema was created to fill gaps in the codebook left by existing schema (see eTable 2 in Supplement 1). A cannabinoid classification schema was then developed based on an extensive search of the existing literature on cannabinoids and review of data by 2 coders (M.C.N. and M.Z.L.).

#### Cannabinoid Classification

Cannabinoid characteristics were clustered and organized into 10 primary classes based on the existing literature: cannabichromenes, cannabicyclols, cannabidiols, cannabielsoins, cannabigerols, cannabinols and cannabinodiols, cannabitriols, Δ^8^-THC, Δ^9^-THC, and other cannabinoids (ie, cannabinoids that did not meet one of the specific classifications listed above).^36,37,38,39^ A total of 111 individual cannabinoid constituents were identified through keywords and abbreviated keywords. Cannabinoid constituency descriptors were identified and assigned verbatim into cannabinoid types and then classified into cannabinoid classes.

#### Cannabis Product Classification

Product classifications were clustered into 5 ROAs (defined as the physiological organ system through which a cannabis product is absorbed into the body): digestive (eg, beverage and solid food), epidermal (eg, diluted agents and direct agents), multisystem (eg, concentrates, plants, and other), oral (eg, tablets), and respiratory (eg, cannabis electronic delivery systems [CEDS], cannabis combustible products [CCP]). Nonconsumable products had one product class—equipment (71 802 [14.33%])—for which there were 2 product categories: accessory (24 111 [33.58%]) and miscellaneous (47 691 [66.42%]). However, this product category was removed from subsequent final analysis and results as products in this class did not contain or use a cannabinoid.

Across ROAs, CDPs were organized into 12 product classes (defined as the medium by which cannabis is delivered to organ systems): beverage, concentrate, diffuse inhalation, direct inhalation, miscellaneous (digestive), other (multisystem), plant, solid (oral), solid food, topical, transdermal, and viscous. Across product categories (groups of similar CDPs based on the assigned product type), 27 groupings were created from product descriptions that included 84 individual product types. Specific product categories, and the product types contained within each category are listed in the eTable 2 in Supplement 1.

#### Cannabis Flavor Classification

Flavor characteristics were organized into 6 primary flavor classes and 33 flavor categories (in parentheses following corresponding flavor class): beverage (alcohol, coffee and tea, dairy or dairy-based drink, flavored drink, and fruit juice), confectionery (bakery, chocolate, composite confectionery, dairy confectionery, grain confectionery, hard candy, ice cream, other candy, soft candy, and sugar), fruit (berry, citrus, melon, tropical, and other fruit); other flavors (inorganic and organic), savory food (composite food, dairy food, grain food, meat, nut, snack, and vegetable), and seasoning (herb, salt, sauce, and spice). Across flavor categories, a total of 260 individual flavor types were identified verbatim from product listings.

## Results

A total of 1 384 845 product listings were identified from the platform, of which 573 854 were unique. As previously stated, we removed 72 842 nonconsumable products that did not contain cannabis for a total of 501 012 product listings that were analyzed with characteristics reported on the platform. Classification, product characteristics, and flavor characteristics of CDP cannabinoids are presented in eFigures 1 and 2 in Supplement 1 (including hyperlinks to interactive visualizations).

### Cannabinoid Classification

Among unique CDP listings, there were 86 408 total mentions of a cannabinoid compound descriptor. The 3 most common cannabinoid compound classes were Δ^9^-THC (54 699 [63.30%]), cannabidiols (22 139 [25.62%]), and cannabigerols (4215 [4.88%]) (see Table 1 for complete breakdown).

**Table 1.** Cannabinoid Compound Descriptors Detected on Weedmaps, US, September 2023 to November 2023

Cannabinoid class and constituency	Cannabinoid compound descriptor detected, No. (%) (N = 86 408)
Cannabichromenes	1141 (1.32)
Cannabichromene	1077 (94.39)
Cannabichromenic acid	64 (5.61)
Cannabichromevarin	Not detected
Cannabichromevarinic	Not detected
Cannabicyclols	134 (0.16)
Cannabicyclol	134 (100)
Cannabicyclovarin	Not detected
Cannabicyclolic acid	Not detected
Cannabidiols	22 139 (25.62)
Cannabidiol	21 461 (96.94)
Cannabidiolic acid	561 (2.53)
Cannabidivarin	111 (0.50)
Cannabidivarinic acid	6 (0.03)
Cannabidiorcol	Not detected
Cannabidiol monomethylether	Not detected
Cannabidiphorol	Not detected
Cannabigerols	4215 (4.88)
Cannabigerol	3488 (82.75)
Cannabigerolic acid	727 (17.25)
Cannabigerolic acid monomethylether	Not detected
Cannabigerovarin	Not detected
Cannabigerovarinic acid	Not detected
Cannabinols and cannabinodiols	3876 (4.49)
Cannabinol	3866 (99.74)
Cannabinolic acid	10 (0.26)
Cannabinodiol	Not detected
CannabinodIvarin	Not detected
Cannabinol methylether	Not detected
Cannabiorcool	Not detected
Cannabinol-C2	Not detected
Cannabinol-C4	Not detected
Cannabivarin	Not detected
Δ^8^-tetrahydrocannabinols	192 (0.22)
Δ^8^-tetrahydrocannabinol	192 (100)
Δ^8^-tetrahydrocannabinolic acid	Not detected
Δ^9^-tetrahydrocannabinols	54 699 (63.30)
Δ^9^-tetrahydrocannabinol	53 955 (98.64)
Δ^9^-tetrahydrocannabivarin	699 (1.28)
Δ^9^-tetrahydrocannabivarinic acid	45 (0.08)
Δ^9^-tetrahydrocannabinol-C4	Not detected
Δ^9^-tetrahydrocannabinolic acid A	Not detected
Δ^9^-tetrahydrocannabinolic acid B	Not detected
Δ^9^-tetrahydrocannabinolic acid-C4	Not detected
Δ^9^-tetrahydrocannabiorcol	Not detected
Δ^9^-tetrahydrocannabiorcolic acid	Not detected
Other cannabinoids	12 (0.01)
Cannbicitran	12 (100)
Cannabielsoins	Not detected

### Cannabis Product Characteristics

Multisystem was the most common ROA for CDPs (205 637 [41.04%]), followed by respiratory (185 296 [36.98%]), digestive (98 941 [19.75%]), epidermal (9487 [1.89%]), and oral (1651 [0.33%]) (see eFigure 1 in Supplement 1). Within the multisystem ROA, product classes included concentrate (89 264 [43.41%]), plant (116 321 [56.57%]), and other (52 [0.03%]). Product categories within the concentrate product class included solvent concentrate (51 553 [25.07%]), solventless concentrate (18 703 [9.10%]), and other concentrate (19 008 [9.24%]). The plant product class had 2 product categories: harvested product (116 032 [56.57%]) and preharvest product (289 [0.14%]). The other product class only continued animal care that included tincture (52 [0.03%]). For the epidermal ROA, product classes were topical (9232 [97.31%]) and transdermal (255 [2.69%]). Product categories within the topical class were diluted application (2257 [23.79%]) and direct application products (6975 [73.52%]). Transdermal product categories included only adhesive products (255 [2.69%]). The digestive ROA included the product classes solid food (79 601 [80.45%]), beverage (14 070 [14.22%]), viscous (3126 [3.16%]), and miscellaneous (1944 [1.96%]). Respiratory product classes included diffuse inhalation products, which included product categories of combusted products (eg, candles) (182 [0.10%]), and direct inhalation product such as CEDS (eg, vapes and cartridges) (110 906 [59.85%]) and CCP (eg, prerolls and joints) (74 208 [40.05%]). Oral product class only contained tablets that included buccal (1651 [0.33%]).

### Cannabis Flavor Characteristics

CDPs that were characterized with at least 1 flavor made up 210 575 (42.03%) of product listings. In total, there were 247 762 instances of flavors across all products (including listings with >1 flavor for the same product); the 3 most common flavors were lemon (22 106 [8.92%]), cake (19 463 [7.86%]), and strawberry (13 961 [5.63%]). Among 260 unique flavor types, 227 039 flavors were grouped into 6 parent flavors classes: beverage (1927 [0.85%]), confectionery (47 081 [20.74%]), fruit (149 640 [65.91%]), other flavors (6261 [2.76%]), savory food (8359 [3.68%]), and seasoning (13 771 [6.07%]) (see Table 2; for a complete breakdown, see eTable 3 in Supplement 1).

**Table 2.** Flavor Descriptors Among Cannabis Derived Products, US, September to November 2023^a^

Flavor class by category flavor	No. (%) (N = 227 039)^b^
Beverages (n = 1927 [0.85%])	
Alcohol	286 (14.84)
Coffee or tea	549 (28.49)
Dairy drink	48 (2.49)
Flavored drink	967 (50.18)
Fruit juice	78 (4.05)
Confectionery (n = 47 081 [20.74%])	
Hard candy	17 (0.4)
Soft candy	960 (2.04)
Other candy	419 (0.89)
Bakery	19 519 (41.46)
Chocolate	11 929 (25.34)
Ice cream	10 778 (22.89)
Dairy confectionery	2069 (4.39)
Grain confectionery	20 (0.04)
Composite confectionery	109 (0.23)
Sugar	3151 (6.69)
Savory food (n = 8359 [3.68%])	
Nut	2853 (34.13)
Vegetable	3318 [39.69])
Dairy food	2125 (25.42)
Grain food	26 (0.31)
Meat	37 (0.44)
Composite food	13 (0.16)
Snack	2 (0.02)
Fruit (n = 149 640 [65.91%])	
Berry	37 646 (25.16)
Citrus	40 511 (27.07)
Melon	11 351 (7.59)
Tropical	25 979 (17.36)
Other fruit	49 518 (33.09)
Other flavors (n = 6261 [2.76%])	
Inorganic	5213 (83.26)
Organic	1052 (16.80)
Seasoning (n = 13 771 [6.07%])	
Sauce	366 (2.66)
Spice	2329 (16.91)
Herb	11 268 (81.82)
Salt	2 (0.01)

^a^ For a complete breakdown of categories, see eTable 3 in Supplement 1.

^b^ Flavor classes may not add up equally to flavor categories since multiple flavors reported can occur simultaneously. Flavor categories may not add up equally to individual flavor type since multiple flavors reported can occur simultaneously. Results do not include non-consumable products as they do not contain cannabis.

CDPs in the respiratory ROA had the highest proportion of products with a characterizing flavor (71 142 [33.78%] of 210 575) among all ROAs. The mean (SD) number of flavors for CDP listings with characterizing flavor descriptions was 1.186 (0.570). Overall, product types with the greatest proportion of flavored products were flower buds (36 458 [7.28%]), cartridge (28 645 [5.72%]), and gummies (25 817 [5.15%]) (see eTable 4 in Supplement 1).

## Discussion

From over half a million unique listings on a popular e-commerce platform, a large diversity of CDP types was detected, including myriad product forms (eg, CCPs, concentrates, CEDS) and ways to consume them (eg, epidermal agents, cooking ingredients for cannabis-infused foods, scented candles). Additionally, approximately 2 of every 5 products included at least 1 characterizing flavor, with 260 total unique flavors detected including those that appeal to youths such as berry, candy, chocolate, and alcohol.

Prior cannabis studies have examined the cannabis market using state retail data from cannabis dispensaries (eg, Colorado, Oregon), reviewed product listings from select online cannabis dispensary websites, analyzed pricing data associated with specific CDPs, and explored cannabis marketing exposure and purchasing behavior from survey instruments, social media, and cannabis e-commerce platform data.^6,23,27,30,40,41,42,43,44^ To our knowledge, this is the first study to conduct comprehensive product surveillance to construct a taxonomy of cannabis products for sale on a nationwide e-commerce dataset. The large number of overall product listings, the diversity of product types, and a high presence of appealing product features (eg, choice of flavoring, types of cannabinoid compounds, and multiple ways to consume cannabis) point to the need for better understanding of how the cannabis product marketplace is evolving in a fragmented regulatory landscape.

Results raise concerns about health policy implications, including initiation of product use among underage youths encouraged by direct-to-consumer advertising promoted via e-commerce sites (eg, the platform we used did not actively use age verification requirements on its main launch page at the time of this study, although age verification may be enforced for individual vendor or dispensary pages). In this study, we observed content appealing to youths in product listings and descriptions found in other studies, including a high percentage of multisystem cannabis products (ie, concentrates and extracts that could raise different health and safety concerns, given the potential for residual solvents), cannabis-infused edibles, and numerous flavored product types and combinations.^45,46,47^ These product features are concerning, as numerous studies have shown that other addictive flavored products (eg, e-cigarettes) can lead to increased uptake by youths, and surveys of adolescent cannabis users have found that use of flavored cannabis is common.^45^ Cannabis product and dispensary listings on the platform also enable consumers to connect via social media, further increasing potential market exposure among youths that can lead to increased cannabis use.^48,49,50,51^

In contrast, the potential impact of the increasing variety in product types and flavors among adults may be mixed. Adult consumers of CDPs for medicinal or therapeutic purposes may benefit from different product types that are easier to use or are available through a wide variety of methods of administration (eg, alternatives to smoked cannabis).^52^ On the other hand, an expanding product range may also increase experimentation, initiation, or problematic use among adults who would otherwise not use traditional CDPs. Furthermore, the impact of exposure to different product types and characteristics (eg, flavoring) on concomitant use or transition of use behavior (eg, from cigarette smoking to cannabis use) is not well understood and needs to be assessed in the context of product regulation and harm reduction.^53^

Future studies should proactively monitor the growing physical and virtual cannabis marketplace for aggressive marketing tactics, further product innovation, and new and emerging product that may shift CDP use patterns toward increased harm. Research supporting a better understanding of risk profiles among new and emerging CDPs is also needed, with future cannabis policy considerations including the possibility of establishing differential regulatory approaches among product types based on evidence of consumer risk and safety, such as imposing greater scrutiny on types of ROA that use flavoring (eg, digested vs inhaled flavored cannabis products). Future studies can also build on the product taxonomy developed in this study, while also examining other important topics in cannabis product regulation, such as underage access, the presence of health benefit claims, and product features targeting specific users or demographics.

Recently, the US Drug Enforcement Agency proposed reclassifying cannabis from a Schedule I to a Schedule III drug, which would represent a historic shift in federal regulation and lead to greater easing of restrictions if enacted.^54^ Consequentially, the online cannabis marketplace will continue to evolve and expand as the policy environment shifts toward legalization.

### Limitations

Limitations of this study include lack of generalizability to the entire cannabis retail marketplace, such as licensed dispensaries not listed on the platform or CDPs offered for sale on other websites. Moreover, based on the data collection approach used for this study, it is possible that not all product listings were collected for the study period, as products may have been removed or added after a particular US region or area of the grid-based map search was completed. We attempted to mitigate this by conducting multiple rounds of data collection where we did not observe immediate differences in product sampling, although we cannot say for certain that all products on the platform were collected without access to underlying commercial data that were not available. Additionally, products detected during the study time frame may not reflect seasonal differences in the platform’s cannabis inventory. Furthermore, we did not conduct statistical testing due to concerns of nonindependence of product characteristics at different levels (eg, state, brand, and dispensary differences) and its potential impact on results. Future studies should attempt to use robust statistical testing methods (eg, multilevel models) to assess potential differences among product characteristics observed and other factors. Despite these limitations, the platform remains one of the largest and most popular cannabis online marketplaces and is an important source of publicly available data on cannabis product listings and marketing content that can inform future actions by public health professionals, policymakers, and regulators.^31,55^

## Conclusions

In this study of more than 500 000 CDPs, we found that nearly half of products were flavored, more than two-fifths had a multisystem ROA, and nearly two-thirds had Δ^9^-THC as their primary cannabinoid compound. This qualitative study shows that the current patchwork of cannabis policies poses challenges to effective regulation of CDPs. The online cannabis marketplace will continue to evolve and expand as the policy environment shifts toward legalization. Similar to other addictive products, existing, new, and emerging CDP characteristics should be properly monitored and assessed for possible regulation and appropriate restrictions to ensure current and future safe and responsible adult use of cannabis.

## Notes

Supplement 1. **eMethods.** Data Collection **eTable 1.** Codebook for Cannabis-Derived Product Flavor Classes, Categories, and Types **eTable 2.** Codebook for Cannabis-Derived Product Routes of Administration and Product Classes, Categories, and Types **eTable 3.** Flavor Descriptors Among Cannabis Derived products, US, September to November 2023 (Full Table) **eTable 4.** Flavor Characteristics of Cannabis-Derived Products on Weedmaps by Route of Administration, US, September 2023 to November 2023 **eFigure 1.** Product Wheel Including Product Type **eFigure 2.** Cannabis Flavor Wheel
eReferences.

Supplement 2.
Data Sharing Statement
