# Supplementary material for: Cannabis-Derived Product Types, Flavors, and Compound Types From an E-Commerce Website
Source: JAMA Netw Open. 2024 Oct 21;7(10):e2440376. doi: 10.1001/jamanetworkopen.2024.40376 (PMC11581530; doi:10.1001/jamanetworkopen.2024.40376)
Supplement: Supplement 1. — eMethods. Data Collection eTable 1. Codebook for Cannabis-Derived Product Flavor Classes, Categories, and Types eTable 2. Codebook for Cannabis-Derived Product Routes of Administration and Product Classes, Categories, and Types eTable 3. Flavor Descriptors Among Cannabis Derived products, US, September to November 2023 (Full Table) eTable 4. Flavor Characteristics of Cannabis-Derived Products on Weedmaps by Route of Administration, US, September 2023 to November 2023 eFigure 1. Product Wheel Including Product Type eFigure 2. Cannabis Flavor Wheel eReferences. [file jamanetwopen-e2440376-s001.pdf]

Supplemental Online Content

Nali MC, Yang JS, Li Z, Larsen MZ, Mackey TK. Cannabis-derived product types, flavors, and compound types from the e-commerce website Weedmaps. *JAMA Netw Open*. 2024;7(10):e2440376. doi:10.1001/jamanetworkopen.2024.40376

eMethods. Data Collection

eTable 1. Codebook for Cannabis-Derived Product Flavor Classes, Categories, and Types

eTable 2. Codebook for Cannabis-Derived Product Routes of Administration and Product Classes, Categories, and Types

eTable 3. Flavor Descriptors Among Cannabis Derived products, US, September to November 2023 (Full Table)

eTable 4. Flavor Characteristics of Cannabis-Derived Products on Weedmaps by Route of Administration, US, September 2023 to November 2023

eFigure 1. Product Wheel Including Product Type

eFigure 2. Cannabis Flavor Wheel

eReferences.

This supplemental material has been provided by the authors to give readers additional information about their work.

© 2024 Nali MC et al. *JAMA Network Open*.

eMethods. Data Collection

eSupplement Appendix

Appendix 1

METHODS

Data Collection

Geolocation approach for CDP listings: For each CDP collected from Weedmaps, Python (version 3.8) was used in combination with reverse geocoder package (version 1.5.1) to identify each product's region of origin on the condition that it was located within the United States. If the coordinate intersects (e.g., ordered pair on a coordinate system where lines intersect and represent common point or location) was located in the United States, the request package (version 2.27.1) was used to enter its latitude and longitude into the search location to gather all products around that specific geolocation.

Example of product attributes data fields collected and extracted from Weedmaps:

Using product IDs, each product's description page was accessed to perform a second round of data mining to collect the entire product description and retailer associated with a product listing. See example below of extracted product listing information.

Product Attribute Data collected from Weedmaps

Product Attribute	Product Attribute Information (example)
• Name<br>• Slug<br>• Variant_slug<br>• Brand<br>• Brand_description<br>• Is_badged<br>• Product_description<br>• Edge_category<br>• Reviews_count<br>• Rating<br>• Favorites_count	• Ganji Gummz 500mg -Blue Razzberry<br>• ganj-gummz-ganj-gummz-500mg-blue-razzberry<br>• gg-500mg-blue-razz-110g<br>• N/a<br>• { OMMA Licensed Edible Company Creators of the Rainbow Nuggz, Ganj Gummz, Rainbow Ropes, Scrappy's, and Surreal Nuggz! }<br>• True<br>• {'-Blue Razzberry flavor -Infused with distillate -Coated in sugar -25 pieces per bag -20mg per piece'}<br>• {'uuid': 'd1934663-b03c-440f-a4fa-a3857d6ec56f', 'name': 'Gummies', 'slug': 'gummies', 'ancestors':

© 2024 Nali MC et al. *JAMA Network Open*.

• Price_stats<br>• Online_ordering<br>• Variant Information	[{'uuid': '3dce0614-7c15-428f-8c32-fd6802d0b75e', 'name': 'Edibles', 'slug': 'edibles'}, {'uuid': 'fd6553ff-34d0-4b9b-a28f-b91d3ed8dc11', 'name': 'Candy', 'slug': 'candy'}]}<br>• 0<br>• 0<br>• 4<br>• {'min': 21.6, 'max': 21.6}<br>• {'deliverable': False, 'pickup': True}<br>• {'slug': 'gg-500mg-blue-razz-110g', 'price': {'id': None, 'unit': 'unit', 'quantity': '1', 'price': 21.6, 'label': 'each', 'on_sale': False, 'original_price': 21.6, 'compliance_net_mg': 105000.0}, 'price_visibility': 'visible', 'price_visibility_title': None, 'price_visibility_description': None, 'listing': {'name': 'Noble Cannabis Co Dispensary', 'wmid': 152824953, 'distance': 74.13295608267269, 'slug': 'noble-cannabis-co-dispensary', 'type': 'dispensary', 'region_id': 1776, 'license_type': 'medical', 'is_brand_preferred_listing': False, 'preferred_organization_position': None}, 'menu_item_id': 128689113, 'aggregate_metrics': {'cbd': 0.0, 'terpenes': 0.0, 'thc': 500.0, 'cbd_unit': '%', 'terpenes_unit': '%', 'thc_unit': 'mg'}, 'current_deal_title': 'SEPT STEALS @ NOBLE CANNABIS CO', 'deal_ids': [922223], 'promo_code': None}

© 2024 Nali MC et al. *JAMA Network Open*.

Data Analysis

eTable 1. Codebook for Cannabis-Derived Product Flavor Classes, Categories, and Types

Flavor Class [1]	Flavor Category (FoodEx2 code) [2]	Flavor Type [3]
Beverage	Alcohol (A06EY)	Beer, Blue Crush, Bourbon, Champagne, Cocktail, Gin, Margarita, Mimosa, Peach Bellini, Pina Colada, Rum, Shirley Temple, Tequila, Whiskey, Vodka, Yuzu Fizz
	Coffee and tea (A04KK, A0D9J, A03JA, A0CZM)	Caffeine, Cappuccino, Coffee, Cold Brew, Espresso, Iced Tea, Lemon Tea, Lemongrass, Matcha, Sweet Tea, Tea, White Tea
	Dairy or dairy-based drink (A0C5S)	Eggnog, Horchata, Milk
	Flavored drink (A0C1T)	Coke, Cola, Cream Soda, Kool-Aid, Fruit Punch, Passion, Punch, Root Beer, Soda, Tropical Punch, Tropicana Punch,
	Fruit juice (A0C1X, A0C1V)	Apple Cider, Cherry Punch, Lemonade, Lem n'Lime, Limeade, Pink Lemonade
Confectionery [4]	Bakery (A0EPQ)	Birthday Cake, Brownie, Cake, Churro, Cookie, Cookie Crumble, Donut, Graham, Graham Cracker, Muffin, Oreo, Pie, Rainbow Chip, Red Velvet, Samoan Cookie, Snickerdoodle, Sugar Cookie, Wedding Cake
	Chocolate (A0C4F)	Chocolate, Chocolate Chip, Coco, Dark Chocolate, Fudge, Milk Chocolate, Mocha, White Chocolate
	Composite Confectionery [5]	Banana Split, Cheesecake, Chocolate S'more, Cookie Dough, Custard, Parfait, Peaches and Cream, S'more, Strawberry Chocolate, Sundae, Tart, Toffee, Sorbet
	Dairy Confectionery (A0C5T)	Butter, Buttercream, Butterscotch, Caramel, Creamer, Dolce, Dulce de Leche, Kool Whip, Salted Caramel, Sweet Cream
	Grain Confectionery (A0EZF, A0EZV)	Biscochito, Biscotti, Cereal, Mochi

© 2024 Nali MC et al. *JAMA Network Open*.

	Hard candy (A06EP)	Buttermint, Candy Cane, Jolly Rancher, Lollipop, Skittles
	Ice cream (A06ES, A0ENY)	Cookies & Cream, Gelato, Ice Cream, Muddy, Rainbow Sherbert, Rocky Road, Sherbert
	Other Candy [6]	Candy, Cotton Candy
	Soft candy (A06EN)	Bubble Gum, Taffy
	Sugar (A0C32)	Confetti, Honey, Marshmallow, Sugar, Nectar
Fruit [7]	Berry	Acai, Berry, Blackberry, Blueberry, Cranberry, Dreamberry, Elderberry, Huckleberry, Kiwi, Loganberry, Marionberry, Passionberry, Pumpkin, Raspberry, Strawberry, Wildberry, Wintergreen
	Citrus	Citrus, Grapefruit, Lemon, Lime, Mandarin, Orange, Tangerine
	Melon	Cantaloupe, Honeydew, Melon, Watermelon
	Tropical	Banana, Coconut, Guava, Mango, Papaya, Passion Fruit, Pineapple, Tiki, Tropical
	Other Fruit	Apple, Apricot, Avocado, Blackcherry, Cherry, Dragon Fruit, Fruit, Grape, Grapeseed, Jungle Fruit, Papaya, Peach, Pear, Plum, Pomegranate
Savory Food	Composite Food [8]	Chicken and Waffle, Chile, Gumbo, Mac n Cheese
	Dairy (A0C5H)	Cheese, Cheddar, Parmesan
	Grain (A0C4B, A0C3V)	Butter'd Bidscuit, Cornbread, Granola, Oat, Oatmeal, Pancake
	Meat (A0C3P)	Bacon, Beef, Chicken, Salmon, Steak
	Nut (A014C)	Almond, Cashew, Hazelnut, Macadamia, Nutter Budder, Nutty Puff'd, Peanut Butter, Pecan, Pistachio
	Snack (A0C1B)	Popcorn, Sour Cream and Onion
	Vegetable (A04JC)	Carrot, Cucumber, Garlic, Onion, Tomato, Ube
Seasoning	Herb (A0F0C)	Basil, Clover, Mint, Peppermint, Rosemary, Spearmint, Watermint
	Salt A0C2R)	Sea Salt
	Sauce (A0C2G)	Apple Butter, BBQ, Buffalo, Honey Butter, Hot Sauce, Ketchup, Maple,

© 2024 Nali MC et al. *JAMA Network Open*.

		Mustard, Pique Hot Sauce, Ranch, Salsa, Sriracha, Syrup, Teriyaki
	Spice (A016S)	Adobo, Black Pepper, Cardamom, Chai, Cinnamon, French Vanilla, Ginger, Moroccan, Truffle, Vanilla, Vanilla Bean
Other Flavors [9]	Inorganic	Alpine Frost, Beach, Charcoal, Assortment, Diesel, Rainbow
	Organic	Aloe, Birchwood, Chamomile, Flower, Grass, Lavender, Leaf, Menthol, Patchouli Suede, Plumeria, Rose, Skunk, Sweet Pea

1. Flavor classes were derived from aggregation of flavor categories into broader groupings of food types. Flavor classes were consistent with European Food Safety Authority (2015) FoodEx2 groupings for the *Beverage* (except for the inclusion of "Dairy or Dairy-Based Drink" flavor category, which was moved from "Dairy Products and Analogues" group in FoodEx2), *Fruit*, and *Seasoning* flavor classes. *Confectionery*, *Savory Food*, and *Other Flavors* flavor classes were created inductively from flavor categories.[1]
2. Flavor categories were created to correspond with European Food Safety Authority FoodEx2 groupings, except for *Fruit* (see Note 7 below) and *Other Flavors*. Corresponding FoodEx2 food codes used for operationalization of food categories are in parentheses.[1]
3. Flavor types were derived verbatim from product listings.
4. The definition of *Confectionery* is from the International Food Information Service (2009) *Dictionary of Food Science and Technology*, *2nd edition* which defines confectioneries as sweetened food products, including sugar confectionery (e.g., sweets, candies, chocolates) and bakery confectionery (bakery products such as cakes and pastries).[2]
5. *Composite Confectionery* is a flavor category inductively created for confectionery products that derive flavor from one or more flavor categories.
6. *Other Candy* is a flavor category that includes candy that does not have a specified hardness (i.e., hard or soft).
7. Flavor categories under the *Fruit* flavor class are derived from Krüsemann et al. (2019) for its emphasis on flavor and not botanical characteristics as is the case with the grouping of *Fruit* in the FoodEx2.[3]
8. The *Composite Food* flavor category is an inductively created grouping for savory food products that derive flavor from one or more flavor categories.
9. *Other Flavors* is a flavor class that was derived inductively to capture flavors not associated with categories listed in the FoodEx2. Inorganic refers to flavors that use descriptors that have no relation to living things; organic refers to flavors that use descriptors not associated with other flavor categories but derive from organic material.[1]

© 2024 Nali MC et al. *JAMA Network Open*.

eTable 2. Codebook for Cannabis-Derived Product Routes of Administration and Product Classes, Categories, and Types

Route of Administration [1]	Product Class [2]	Product Category [3]	Product Type [4]
Digestive [5]	Beverage	Alcohol (A06EY)	Wine, Spirits, Mixed Alcoholic Drink
		Drink mix (A0F0Q)	Beverage Enhancer
		Water-based (A0ENR)	Carbonated Drink, Energy Drink, Fruit-Infused Drink
	Solid food [6]	Animal care [8]	Pet Food
		Bundle	Variety
		Bakery (A0EPQ)	Brownie, Chewy Bars, Cookie, Pie
		Confection (A06EJ)	Candy, Gummies
		Dairy (A06ES, A0ENY)	Ice Cream
		Fruit (A0EZN)	Dry Fruit
		Meat (A0C3P)	Jerky
		Savory Snack (A0C1B)	Small Snacks
	Miscellaneous	Capsules	Hard Shell, Soft Shell
	Viscous [7]	Sugar (A0C31)	Syrup
		Oils (A0EMP, A06ET)	Cooking Lubricants
Epidermal	Topical	Direct application	Balms, Salves, Creams, Gel, Lotion, Mask, Ointment, Tincture, Toner, Lubricant
		Diluted application [9]	Bath Bomb, Bath Essentials, Essential Oil, Soaking Salt
	Transdermal	Adhesive	Patches
Multisystem	Concentrate [10]	Solvent concentrate	Badder, BHO, Budder, Crumble, Crystalline, Cured Resin, Cured Sugar, Live Resin, Nug Run, Sauce, Shatter, Sugar, Wax
		Solventless concentrate	Dry Sift, Hash, Ice Hash, Live Rosin, Live Sugar, Live Wet Diamond, Rosin
		Other concentrate	Distillate, Sugar Leaf, Syringe, Variety
	Plant [11]	Pre-harvest	Clone, Seeds, Whole Plant
		Harvested	Buds, Shake, Trim
	Other	Animal care	Tincture
Oral	Solid	Tablet	Buccal
Respiratory [12]	Diffuse inhalation [13]	Combusted	Candle

© 2024 Nali MC et al. *JAMA Network Open*.

	Direct inhalation	Cannabis electronic delivery system	Cartridge, Disposable, Pen, Pods, Stick Battery, Vape, Vaporizer
		Processed cannabis combustible product	Blunt, Cigar, Infused Pre-roll, Joint, Hemp Roll Wraps, Leaf Roll Wraps, Unspecific Wraps

1. Routes of Administration (RoA) was established as the first level of categorization since no existing singular product classification system exists for all cannabis products. Thus, Routes of Administration groupings were created to simplify lower-level groupings and ensure consistency among product classes and categories. Routes of administration are defined as the primary physiological organ system through which a cannabis product is absorbed into the body.
2. Product classes refer to the broad categories of mediums by which cannabis is delivered to organ systems. They are specific to each route of administration and may not be comparable across routes of administration. Product classes for *Epidermal*, *Multisystem*, and *Oral* routes of administration were developed inductively while those for *Digestive* and *Respiratory* routes of administration were adapted from other sources (see notes 5 and 12 below).
3. Within each product class, cannabis derived products (CDPs) were grouped into product categories which delineated the specific product form in the retail marketplace.
4. Product types were derived verbatim from product listings.
5. Product categories for the *Digestive* route of administration are correlated with groupings in the European Food Safety Authority (2009) FoodEx2 classification system as indicated by the FoodEx2 code in parentheses.[1]
6. The *Solid Food* product class includes CDPs colloquially referred to as “edibles.”
7. The *Viscous* product class includes non-solid, non-water-based products that have high viscosity when in the form in which they are purchased and stored.
8. The *Animal Care* product type includes CDPs created for animal consumption or care.
9. *Diluted Application* refers to CDPs which are first diluted in water prior to contact with skin. This contrasts with *Direct Application* CDPs in which the product is applied directly to the skin.
10. *Concentrate* refers to CDPs that are derived from distilling a part of the cannabis plant. Solvent and solventless concentrates refer to CDPs that are produced using different extractive methods, either using or not using a solvent in the distillation process.
11. The *Plant* product class refers to botanical CDPs in various stages of the growth and harvesting process. The *Pre-Harvest* product category refers to the cannabis plant in any stages through flowering. The *Harvested Product* category refers to cannabis flowers that have been harvested from the plant but not yet processed in any other way.
12. Product categories for the *Respiratory* route of administration were adapted from the work of Spindle et al. (2019).[4]
13. *Diffuse Inhalation* refers to CDPs that are first diffused into the ambient air before being inhaled. This contrasts with *Direct Inhalation* CDPs in which inhalation of an emission produced by a CDP occurs directly from the product.

Terpenes sub-analysis

A comprehensive keywords filtering process was conducted on the product descriptions to identify references to terpenes. This process identified a total of 15,070 CDP listings that included terpene-related descriptions. However, it is important to note that these terpenes detected were not classified as flavor characteristics. Instead, the primary focus of these descriptions was on the additional flavoring agents analyzed. The distinction between terpenes as functional ingredients versus flavor enhancers was carefully removed from the primary analysis.

© 2024 Nali MC et al. *JAMA Network Open*.

eTable 3: Flavor Descriptors Among Cannabis Derived products, US, September to November 2023 (Full Table)

Flavor class by category and Individual Flavor	No. (%) (N= 227 039)[a]
**Beverages (n= 1927 [0.85%])**	
Alcohol	***(n= 286 [14.84])***[b]
Blue Crush	(n=1, 0.0004%)
Bourbon	(n=2, 0.0008%)
Champagne	(n=62, 0.025%)
Cocktail	(n=1, 0.0004%)
Gin	(n=2, 0.0008%)
Margarita	(n=57, 0.023%)
Mimosa	(n=61, 0.025%)
Peach Bellini	(n=1, 0.0004%)
Pina Colada	(n=82, 0.033%)
Rum	(n=2, 0.0008%)
Shirley Temple	(n=2, 0.0008%)
Tequila	(n=6, 0.0024%)
Whiskey	(n=2, 0.0008%)
Vodka	(n=3, 0.0012%)
Yuzu Fizz	(n=1, 0.0004%)
Coffee or Tea	***(n=549 [28.49])***[b]
Caffeine	(n=3, 0.0012%)
Cappuccino	(n=1, 0.0004%)
Coffee	(n=381, 0.154%)
Cold Brew	(n=1, 0.0004%)
Espresso	(n=7, 0.0028%)
Iced tea	(n=2, 0.0008%)
Lemon Tea	(n=1, 0.0004%)
Lemongrass	(n=5, 0.002%)
Matcha	(n=51, 0.021%)
Sweet Tea	(n=1, 0.0004%)
Tea	(n=94, 0.038%)
White Tea	(n=1, 0.0004%)
Dairy drink	***(n=48 [2.49])***[b]
Eggnog	(n=1, 0.0004%)
Horchata	(n=5, 0.002%)
Milk	(n=42, 0.017%)
Flavored drink	967 (50.18)
Cherry Cola	(n=1, 0.0004%)
Coke	(n=5, 0.002%)
Cola	(n=267, 0.11%)
Cream Soda	(n=3, 0.0012%)
Kool-Aid	(n=2, 0.0008%)
Lemonade	(n=50, 0.02%)
Lem n'Lime	(n=2, 0.0008%)
Limeade	(n=13, 0.005%)

© 2024 Nali MC et al. *JAMA Network Open*.

Pink Lemonade	(n=8, 0.003%)
Root Beer	(n=225, 0.091%)
Soda	(n=130, 0.052%)
Fruit Juice	***(n=78 [4.05])[b]***
Apple Cider	(n=3, 0.0012%)
Cherry Punch	(n=1, 0.0004%)
Fruit Punch	(n=16, 0.0065%)
Passion	(n=27, 0.011%)
Punch	(n=288, 0.116%)
Tropical Punch	(n=5, 0.002%)
Tropicana Punch	(n=1, 0.0004%)
**Confectionery (n=47 081 [20.74%])**	
Hard candy	***(n=17 [0.4])[c]***
Buttermint	(n=6, 0.002%)
Candy Cane	(n=5, 0.002%)
Jolly Rancher	(n=4, 0.002%)
Lollipop	(n=1, 0.0004%)
Skittles	(n=1, 0.0004%)
Soft candy	***(n=960 [2.04])[c]***
Bubble Gum	(n=945, 0.38%)
Taffy	(n=6, 0.002%)
Other candy	***(n=419 [0.89])[c]***
Candy	(n=18, 0.007%)
Cotton Candy	(n=401, 0.162%)
Bakery	***(n=19,519 [41.46])[c]***
Birthday Cake	(n=15, 0.006%)
Brownie	(n=7, 0.003%)
Cake	(n=19,463, 7.86%)
Churro	(n=20, 0.008%)
Cookie	(n=3, 0.0012%)
Cookie Crumble	(n=1, 0.0004%)
Donut	(n=32, 0.013%)
Graham	(n=1, 0.0004%)
Graham Cracker	(n=1, 0.0004%)
Muffin	(n=1, 0.0004%)
Oreo	(n=21, 0.008%)
Pie	(n=5, 0.002%)
Rainbow Chip	(n=4, 0.002%)
Red Velvet	(n=19, 0.008%)
Samoan Cookies	(n=1, 0.0004%)
Snickerdoodle	(n=37, 0.0150%)
Sugar Cookie	(n=1, 0.0004%)
Wedding Cake	(n=2, 0.0008%)
Chocolate	***(n=11,929 [25.34])[c]***
Chocolate	(n=11483, 4.63%)
Chocolate Chip	(n=184, 0.074%)
Coco	(n=13, 0.0052%)
Dark Chocolate	(n=14, 0.0057%)
Fudge	(n=216, 0.087%)

© 2024 Nali MC et al. *JAMA Network Open*.

Milk Chocolate	(n=3, 0.0012%)
Mocha	(n=127, 0.051%)
White Chocolate	(n=16, 0.0065%)
Ice cream	***(n=10,778 [22.89])[c]***
Banana Split	(n=1, 0.0004%)
Cheesecake	(n=22, 0.009%)
Chocolate S'mores	(n=1, 0.0004%)
Cookie Dough	(n=8, 0.003%)
Custard	(n=1, 0.0004%)
Parfait	(n=3, 0.0012%)
Peaches and Cream	(n=1, 0.0004%)
S'more	(n=10, 0.004%)
Strawberry Chocolate	(n=1, 0.0004%)
Sundae	(n=30, 0.012%)
Tart	(n=18, 0.007%)
Toffee	(n=7, 0.003%)
Sorbet	(n=6, 0.002%)
Dairy confectionery	***(n=2069 [4.39])[c]***
Butter	(n=64, 0.026%)
Buttercream	(n=1, 0.0004%)
Butterscotch	(n=133, 0.054%)
Caramel	(n=1890, 0.763%)
Creamer	(n=2, 0.0008%)
Dolce	(n=1, 0.0004%)
Dulce de Leche	(n=3, 0.0012%)
Kool Whip	(n=1, 0.0004%)
Salted Caramel	(n=2, 0.0008%)
Sweet Cream	(n=3, 0.0012%)
Grain confectionery	***(n=20 [0.04])[c]***
Biscochito	(n=2, 0.0008%)
Biscotti	(n=2, 0.0008%)
Cereal	(n=9, 0.0036%)
Mochi	(n=7, 0.003%)
Composite confectionery	***(n=109 [0.23])[c]***
Cookies & cream	(n=944, 0.381%)
Gelato	(n=9766, 3.94%)
Ice Cream	(n=33, 0.013%)
Muddy	(n=2, 0.0008%)
Rainbow Sherbert	(n=2, 0.0008%)
Rocky Road	(n=1, 0.0004%)
Sherbert	(n=63, 0.025%)
Sugar	***(n=3151 [6.69])[c]***
Confetti	(n=9, 0.0036%)
Honey	(n=3134,1.26%)
Marshmallow	(n=6, 0.002%)
Sugar	(n=9, 0.0036%)
Nectar	(n=1, 0.0004%)
**Savory food (n=8359 [3.68%])**	
Nut	***(n=2853 [34.13])[d]***

© 2024 Nali MC et al. *JAMA Network Open*.

Almond	(n=12, 0.0048%)
Cashew	(n=3, 0.0012%)
Hazelnut	(n=7, 0.0028%)
Macadamia	(n=15, 0.006%)
Nutter Budder	(n=1, 0.0004%)
Nutty Puff'd	(n=1, 0.0004%)
Peanut butter	(n=2742, 1.11%)
Pecan	(n=4, 0.002%)
Pistachio	(n=71, 0.029%)
Vegetable	***(n=3318 [39.69])[d]***
Carrot	(n=21, 0.0085%)
Cucumber	(n=146, 0.059%)
Garlic	(n=3102, 1.25%)
Onion	(n=50, 0.02%)
Tomato	(n=1, 0.0004%)
Dairy food	***(n=2125 [25.42])[d]***
Cheese	(n=2111, 0.85%)
Cheddar	(n=10, 0.004%)
Parmesan	(n=3, 0.0012%)
Grain food	***(n=26 [0.31])[d]***
Butter'd biscuit	(n=1, 0.0004%)
Cornbread	(n=1, 0.0004%)
Granola	(n=4, 0.002%)
Oat	(n=1, 0.0004%)
Oatmeal	(n=18, 0.0073%)
Pancake	(n=1, 0.0004%)
Meat	***(n=37 [0.44])[d]***
Bacon	(n=3, 0.0012%)
Beef	(n=3, 0.0012%)
Chicken	(n=18, 0.0073%)
Salmon	(n=12, 0.0048%)
Steak	(n=1, 0.0004%)
Composite food	***(n=13 [0.16])[d]***
Chicken and Waffle	(n=1, 0.0004%)
Chile	(n=2, 0.0008%)
Gumbo	(n=7, 0.0029%)
Mac n Cheese	(n=3, 0.0012%)
Snack	***(n=2 [0.02])[d]***
Popcorn	(n=1, 0.0004%)
Sour Cream and Onion	(n=1, 0.0004%)
Ube	(n=1, 0.0004%)
**Fruit (n=149 640 [65.91%])**	
Berry	***(n=37,646 [25.16])[e]***
Acai	(n=41, 0.017%)
Berry	(n=5212, 2.10%)
Blackberry	(n=3178, 1.28%)
Blackcherry	(n=1, 0.0004%)
Blueberry	(n=8533, 3.44%)
Cranberry	(n=61, 0.025%)

© 2024 Nali MC et al. *JAMA Network Open*.

Dreamberry	(n=9, 0.0036%)
Elderberry	(n=5, 0.002%)
Guava	(n=3409, 1.38%)
Huckleberry	(n=28, 0.011%)
Kiwi	(n=1146, 0.46%)
Loganberry	(n=1, 0.0004%)
Marionberry	(n=30, 0.012%)
Passionberry	(n=2, 0.0008%)
Passion Fruit	(n=250, 0.101%)
Pumpkin	(n=17, 0.007%)
Raspberry	(n=6134, 2.48%)
Strawberry	(n=13961, 5.63%)
Wildberry	(n=31, 0.013%)
Wintergreen	(n=3, 0.0012%)
Citrus	***(n=40,511 [27.07])***[e]
Citrus	(n=1572, 0.63%)
Grapefruit	(n=1210, 0.49%)
Lemon	(n=22106, 8.92%)
Lime	(n=5819, 2.35%)
Mandarin	(n=66, 0.27%)
Orange	(n=9504, 3.84%)
Tangerine	(n=1095, 0.44%)
Melon	***(n=11,351 [7.59])***[e]
Cantaloupe	(n=45, 0.18%)
Honeydew	(n=19, 0.008%)
Melon	(n=1884, 0.76%)
Plum	(n=205, 0.083%)
Watermelon	(n=9321, 3.76%)
Tropical	***(n=25,979 [17.36])***[e]
Banana	(n=9608, 3.88%)
Coconut	(n=496, 0.20%)
Mango	(n=6308, 2.55%)
Pineapple	(n=7284, 2.94%)
Tiki	(n=1, 0.0004%)
Tropical	(n=3, 0.0012%)
Other fruit	***(n=49,518 [33.09])***[e]
Apple	(n=9293, 3.75%)
Apricot	(n=813, 0.33%)
Avocado	(n=1, 0.0004%)
Cherry	(n=13,359, 5.39%)
Dragon Fruit	(n=7, 0.003%)
Fruit	(n=7252, 2.93%)
Grape	(n=10,597, 4.28%)
Grapeseed	(n=1, 0.0004%)
Jungle Fruit	(n=1, 0.0004%)
Papaya	(n=3, 0.0012%)
Peach	(n=5080, 2.05%)
Pear	(n=1855, 0.75%)
Pomegranate	(n=910, 0.368%)

© 2024 Nali MC et al. *JAMA Network Open*.

**Other flavors (n=6261 [2.76%])**	
Inorganic	***(n=5213 [83.26])[f]***
Alpine Frost	(n=1, 0.0004%)
Beach	(n=1, 0.0004%)
Charcoal	(n=1, 0.0004%)
Skunk	(n=4, 0.002%)
Assortment	(n=5, 0.002%)
Diesel	(n=5200, 2.10%)
Rainbow	(n=1, 0.0004%)
Organic	***(n=1052 [16.80])[f]***
Aloe	(n=4, 0.002%)
Birchwood	(n=2, 0.0008%)
Chamomile	(n=1, 0.0004%)
Flower	(n=1, 0.0004%)
Grass	(n=1, 0.0004%)
Lavender	(n=1042, 0.421%)
Leaf	(n=1, 0.0004%)
Menthol	(n=155, 0.063%)
Patchouli Suede	(n=1, 0.0004%)
Plumeria	(n=1, 0.0004%)
Rose	(n=10, 0.004%)
Sweet Pea	(n=3, 0.0012%)
**Seasoning (n=13 771 [6.07%])**	
Sauce	***(n=366 [2.66])[g]***
Apple Butter	(n=1, 0.0004%)
BBQ	(n=10, 0.004%)
Buffalo	(n=2, 0.0008%)
Honey Butter	(n=1, 0.0004%)
Hot Sauce	(n=2, 0.0008%)
Ketchup	(n=5, 0.002%)
Maple	(n=280, 0.113%)
Mustard	(n=20, 0.008%)
Pique Hot Sauce	(n=1, 0.0004%)
Ranch	(n=9, 0.0036%)
Salsa	(n=1, 0.0004%)
Sriracha	(n=3, 0.0012%)
Syrup	(n=5, 0.002%)
Teriyaki	(n=1, 0.0004%)
Spice	***(n=2329 [16.91])[g]***
Adobo	(n=1, 0.0004%)
Black Pepper	(n=1, 0.0004%)
Cardamon	(n=1, 0.0004%)
Chai	(n=2, 0.0008%)
Cinnamon	(n=696, 0.281%)
French Vanilla	(n=1, 0.0004%)
Ginger	(n=135, 0.055%)
Moroccan	(n=14, 0.0057%)
Truffle	(n=7, 0.0028%)
Vanilla	(n=1500, 0.605%)

© 2024 Nali MC et al. *JAMA Network Open*.

Vanilla Bean	(n=1, 0.0004%)
Herb	***(n=11,059 [81.82])[g]***
Basil	(n=8, 0.0032%)
Clover	(n=1, 0.0004%)
Mint	(n=11,059, 4.46%)
Peppermint	(n=172, 0.069%)
Rosemary	(n=28, 0.011%)
Spearmint	(n=3, 0.0012%)
Watermint	(n=1, 0.0004%)
Salt	***(n=2 [0.01])[g]***
Sea Salt	(n=2, 0.0008%)

*Flavor classes may not add up equally to flavor categories since multiple flavors reported can occur simultaneously. Flavor categories may not add up equally to individual flavor type since multiple flavors reported can occur simultaneously. Results do not include non-consumable products as they do not contain cannabis.
[b] Calculated as a percentage of beverage class.
[c] Calculated as a percentage of confectionery class.
[d] Calculated as a percentage of savory food class.
[e] Calculated as a percentage of fruit class.
[f] Calculated as a percentage of other flavors class.
[g] Calculated as a percentage of seasoning class.

eTable 4. Flavor Characteristics of Cannabis-Derived Products on Weedmaps by Route of Administration, US, September 2023 to November 2023

Route of Administration (RoA)	Total Products	Total Flavored Products	Average Number of Flavors Among all Products (i.e., flavored and non-flavored)	Average Number of Flavors Among Flavor Products Only
**Oral**	1651	193 (0.09%)	0.128	1.098
**Epidermal**	9487	1291 (0.61%)	0.148	1.088
**Digestive**	98,941	67,576 (32.09%)	0.854	1.251
**Respiratory**	185,296	71,142 (33.78%)	0.442	1.152
**Multisystem**	205,637	70,373 (33.41%)	0.395	1.160
**Total**	501,012	210,575	0.498	1.186

© 2024 Nali MC et al. *JAMA Network Open*.

FIGURES:

eFigure 1. Product Wheel Including Product Type

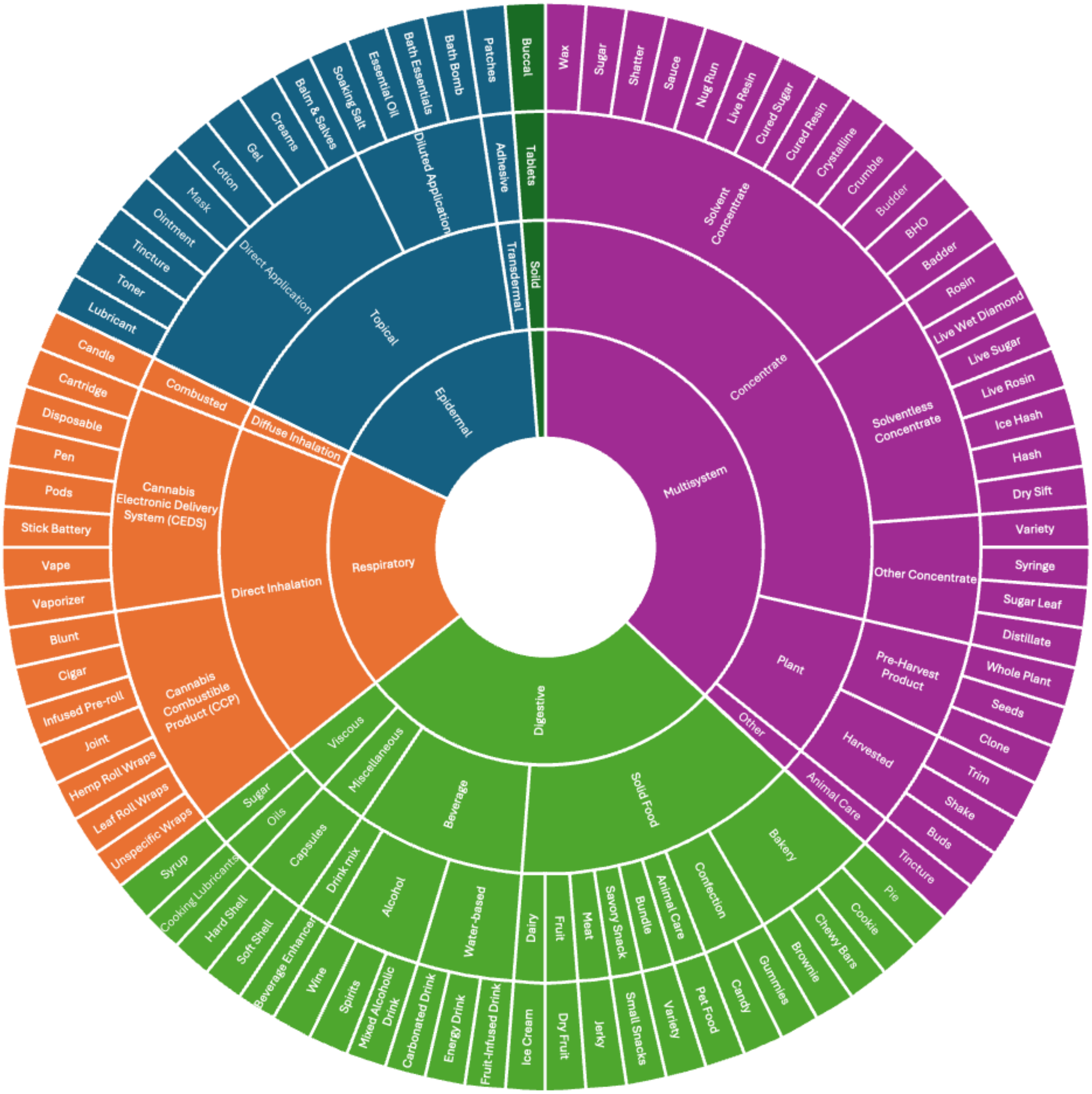

__Figure 1:__ Product Wheel. Innermost layer and broadest level of product grouping is the route of administration (RoA); the second innermost layer is the product class, third innermost layer is the product category, and outer layer is the product type. An interactive version of the wheel can be found at: www.s-3.io/wheels.

© 2024 Nali MC et al. *JAMA Network Open*.

eFigure 2. Cannabis Flavor Wheel

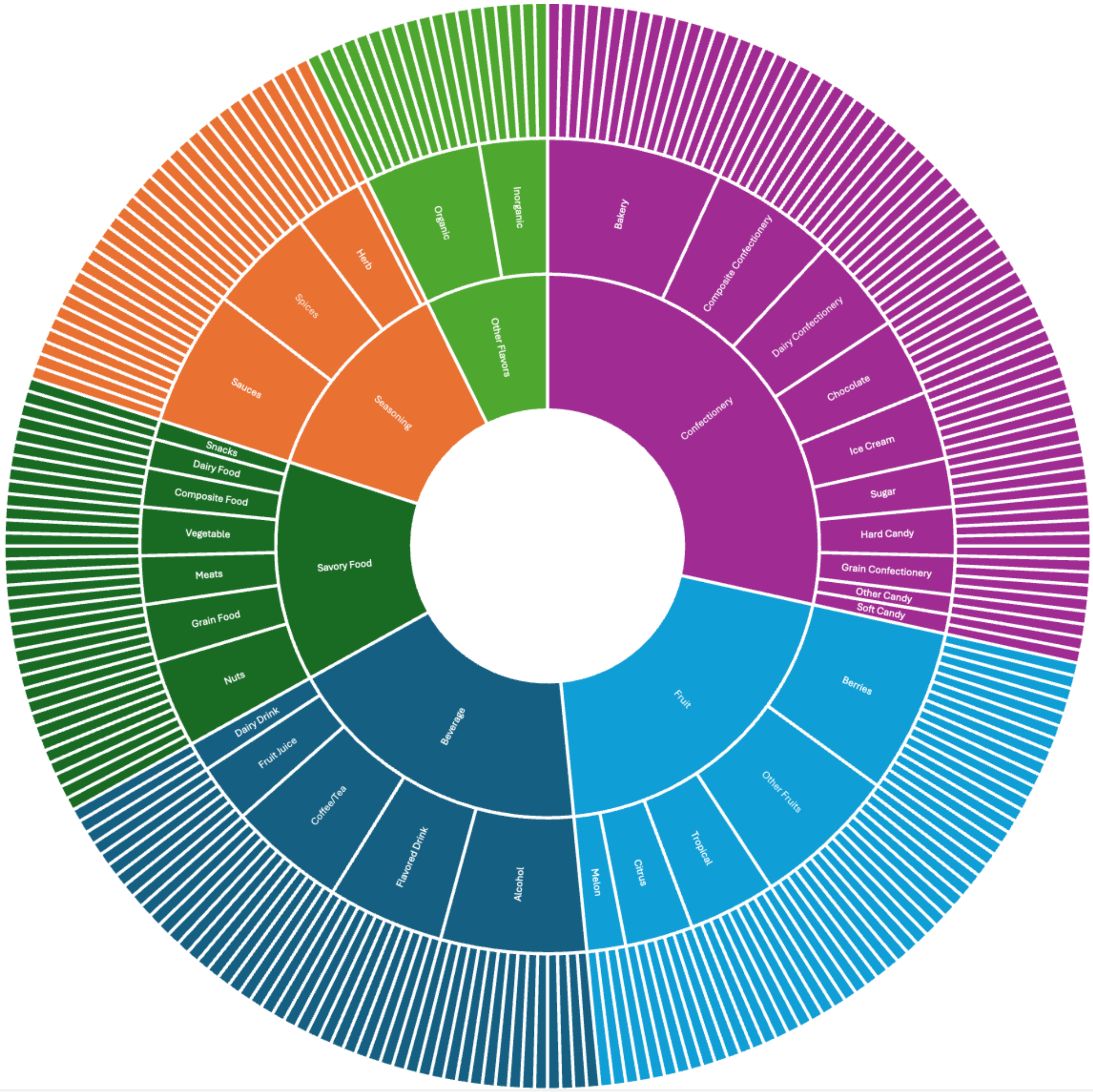

Figure 2: *Cannabis Flavor Wheel. The innermost layer and broadest level of flavor groups is the flavor class; the middle layer is the flavor category, and the outer layer is the flavor type. An interactive version of the wheel can be found at: www.s-3.io/wheels.*

© 2024 Nali MC et al. *JAMA Network Open*.

eReferences

1. The food classification and description system FoodEx 2 (revision 2). EFSA Supporting Publications. 2017;12(5). doi:10.2903/SP.EFSA.2015.EN-804

2. Small E, Cronquist A. A PRACTICAL AND NATURAL TAXONOMY FOR CANNABIS. Taxon. 1976;25(4):405-435. doi:10.2307/1220524

3. Krüsemann EJZ, Boesveldt S, De Graaf K, Talhout R. An E-Liquid Flavor Wheel: A Shared Vocabulary Based on Systematically Reviewing E-Liquid Flavor Classifications in Literature. Nicotine & Tobacco Research. 2019;21(10):1310-1319. doi:10.1093/NTR/NTY101

4. Spindle TR, Bonn-Miller MO, Vandrey R. Changing landscape of cannabis: novel products, formulations, and methods of administration. Curr Opin Psychol. 2019;30:98. doi:10.1016/J.COPSYC.2019.04.002

© 2024 Nali MC et al. *JAMA Network Open*.
