# Supplementary material for: Cannabis-Derived Product Types, Flavors, and Compound Types From an E-Commerce Website
Source: JAMA Netw Open. 2024 Oct 21;7(10):e2440376. doi: 10.1001/jamanetworkopen.2024.40376 (PMC11581530; doi:10.1001/jamanetworkopen.2024.40376)
Supplement: Supplement 2. — Data Sharing Statement [file jamanetwopen-e2440376-s002.pdf]

Data Sharing Statement

Nali. Cannabis-Derived Product Types, Flavors, and Compound Types From the E-Commerce Website Weedmaps. *JAMA Netw Open*. Published October 21, 2024.
doi:10.1001/jamanetworkopen.2024.40376

Data
Data available: Yes
Data types: Data (not involving human participants)
How to access data: De-identified datasets are available upon reasonable request to the corresponding author.
When available: With publication

Supporting Documents
Document types: None

Additional Information
Who can access the data: Researchers requesting data
Types of analyses: For any purpose
Mechanisms of data availability: Without investigator support
Any additional restrictions: Data will be appropriately de-identified from commercial information
